# Dynamics and activity of an ammonia-oxidizing archaea bloom in South San Francisco Bay

**DOI:** 10.1093/ismejo/wrae148

**Published:** 2024-07-30

**Authors:** Anna N Rasmussen, Christopher A Francis

**Affiliations:** Department of Earth System Science, Stanford University, Stanford, CA 94305, United States; Department of Earth System Science, Stanford University, Stanford, CA 94305, United States; Oceans Department, Stanford University, Stanford, CA 94305, United States

**Keywords:** estuary, nitrification, ammonia-oxidizing archaea, nitrite

## Abstract

Transient or recurring blooms of ammonia-oxidizing archaea (AOA) have been reported in several estuarine and coastal environments, including recent observations of AOA blooms in South San Francisco Bay. Here, we measured nitrification rates, quantified AOA abundance, and analyzed both metagenomic and metatranscriptomic data to examine the dynamics and activity of nitrifying microorganisms over the course of an AOA bloom in South San Francisco Bay during the autumn of 2018 and seasonally throughout 2019. Nitrification rates were correlated with AOA abundance in quantitative polymerase chain reaction (PCR) data, and both increased several orders of magnitude between the autumn AOA bloom and spring and summer seasons. From bloom samples, we recovered an extremely abundant, high-quality *Candidatus* Nitrosomarinus catalina-like AOA metagenome-assembled genome that had high transcript abundance during the bloom and expressed >80% of genes in its genome. We also recovered a putative nitrite-oxidizing bacteria metagenome-assembled genome from within the *Nitrospinaceae* that was of much lower abundance and had lower transcript abundance than AOA. During the AOA bloom, we observed increased transcript abundance for nitrogen uptake and oxidative stress genes in non-nitrifier metagenome-assembled genomes. This study confirms AOA are not only abundant but also highly active during blooms oxidizing large amounts of ammonia to nitrite—a key intermediate in the microbial nitrogen cycle—and producing reactive compounds that may impact other members of the microbial community.

## Introduction

Nitrification is a key step in the microbial nitrogen (N) cycle whereby ammonia is converted to nitrate, thus linking N-fixation (and decomposition of organic N) to N-loss processes via a generally tightly coupled two-step process consisting of ammonia and then nitrite oxidation. Several guilds of microorganisms are responsible for carrying out nitrification, including ammonia-oxidizing archaea (AOA) and bacteria (AOB), nitrite-oxidizing bacteria (NOB), and comammox bacteria. The ecology and activity of nitrifiers is of particular interest in systems polluted by excess ammonia such as most estuaries. San Francisco Bay (SFB)—a large and biologically, economically, and socially vital estuary—receives high loads of ammonia from dozens of wastewater treatment plants (WWTPs) [1] serving the ~8 million human inhabitants of the “Bay” Area. Recent DNA-based studies revealed a previously undocumented AOA bloom occurring in South SFB [2, 3]. The biogeochemical and transcriptional activity of these AOA and other microbial community members during the bloom is the focus of this study.

In estuarine waters, the predominance of AOA or AOB often depends on several factors, most commonly salinity, particle association, nutrient status, and dissolved oxygen [4–11]; however, AOA are often numerically dominant in the free-living fraction and generally derived from the family *Nitrosopumilaceae* [6, 8, 9, 12]. Of particular interest, AOA have been documented to form seasonal blooms in summer [13], autumn [14], and winter [15] in coastal environments or transient blooms after storm [10], wind [16], or other physical disturbances [17]. In many of these examples, AOA blooms have led to nitrite accumulation in waters, both oxic and suboxic. Perhaps the best studied AOA bloom is that off Sapelo Island, GA, USA, in warm, oxic, brackish waters. Metatranscriptomes from Sapelo Island reveal that AOA are extremely active during the bloom and genes related to general metabolism such as ammonia oxidation and carbon fixation are most highly transcribed [13]. In two bays of the Yellow Sea with near marine salinities, AOA and MGIIb *Euryarchaea* were more abundant than bacterial phyla in 16S rRNA gene amplicon data in October 2015 [14]. AOA reached ~30% and *Euryarchaea* reached ~20% relative abundance of the overall bacterial and archaeal community in both the Garorim and Gyeonggi Bays in autumn, suggesting *Euryarchaea* may support AOA bloom formation [14]. We recently described recurring massive AOA blooms in South SFB leading to nitrite accumulation in oxic, poly- to euhaline waters most autumns from 2012 to 2020 [2]. In 16S rRNA gene amplicon data from 2012 to 2014, AOA reached ~20% relative abundance in the bacterial and archaeal community in contrast to NOB, which were of very low relative abundance during AOA blooms [2]. Unlike in the Yellow Sea, we did not find evidence of a concomitant *Euryarchaea* bloom [2]. We recovered a high-quality *Candidatus* Nitrosomarinus catalina metagenome-assembled genome (MAG) from 2013 AOA bloom samples and found that this bloom AOA genome along with other *Ca.* Nitrosomarinus genomes are small, have high coding density, and low GC content [2]. Although *Ca.* Nitrosomarinus genomes are streamlined, most contain urease genes that could allow for the use of urea as an alternative source of ammonia during low-ammonia conditions such as during the AOA bloom in SFB [2].

Here, we explore the dynamics of the massive AOA blooms in South SFB, with the goal of answering a number of key research questions. What are the nitrification rates during these massive AOA blooms? Are NOB active despite low abundances? Do other microbes show high transcriptional activity during the bloom? Can we identify potential interactions with AOA and other members of the microbial community? We use ^15^N-based stable isotope incubations to measure nitrification rates for South SFB, combined with quantitative PCR (qPCR), metagenomics, and metatranscriptomics, to understand links between biogeochemical rates and the microbial community. We sampled from October 2018 to December 2019, capturing a large AOA bloom in autumn of 2018.

## Materials & methods

### Sample collection

Sampling took place at Station 27 (Fig. S1) between October 2018 and December 2019 during United States Geological Survey (USGS) Water Quality monitoring cruises in the main channel of the SFB estuary onboard the *R/V Peterson*. We sampled five times from October to December 2018 and seasonally (February, May, July, and December) in 2019 (Fig. S2). Water was collected from shallow (2 m) flow-through and bottom waters (1 m above estuary floor) via Niskin bottle casts and prefiltered through an 80 μm pore size mesh. Microbial biomass was then collected by filtering 150–1000 ml of water through a 0.22 μm polyethersulfone Supor-200 membrane filter (47 mm diameter; Pall, Port Washington, NY), followed by flash-freezing the filter in liquid nitrogen prior to storage at −80°C.

### Environmental data

Water quality data were measured by USGS and downloaded from the USGS Water Quality of SFB database [18]. Additional ammonium, nitrate, and nitrite measurements were performed on filtered (0.22 μm pore size) water as previously described [2]. For samples with ammonia concentrations below the limit of detection using the salicylate–hypochlorite method [19] (<0.5 μM), ammonia was additionally measured using a fluorometric method [20]; however, some samples still had undetectable ammonia concentrations (<0.01 μM).

### Stable isotope incubations

Nitrification rates were measured following previously described methods [21, 22]. Briefly, stable isotope incubations were set up in opaque gas sampling bags with 450 ml of 80 μm-prefiltered Bay water and sampled after initial (T0) addition of ^15^N-ammonium and after 6 h (T6) of incubation. ^15^N-NO_x_ in both T0 and T6 subsamples was measured by the University of California Davis Stable Isotope Facility (Davis, CA) using the denitirifier method [23] to convert ^15^N-NO_x_ to ^15^N-N_2_O, which was then measured via isotope-ratio mass spectrometry (https://stableisotopefacility.ucdavis.edu/nitrate-no3-water). Based on atom fraction, rates were then calculated using an endpoint model to estimate flux from the ammonium to NO_x_ (nitrate + nitrite) pool as described in [21]. All incubations were performed in dark conditions; bottom water samples were all taken well below the photic zone, whereas shallow (2 m) samples ranged from outside the photic zone (<1% surface irradiance) to 20% surface irradiance (median = 9%; see Fig. S2). See Supplemental Materials & Methods for detailed description of setup and rate calculations.

### DNA extraction

DNA and RNA were extracted using a phenol co-extraction method modified [24] and [25] (as detailed in the Supplemental Materials & Methods).

### Quantitative PCR analysis of Marine Group I Thaumarchaeota 16S rRNA gene abundance

qPCR was performed on microbial DNA samples using a fluorescent TaqMan assay amplifying a region of the 16S rRNA gene of Marine Group I (MGI) Thaumarchaeota (representing AOA abundance) [26], using primers GI_751F (GTCTACCAGAACAYGTTC) and GI_956R (HGGCGTTGACTCCAATTG) and TaqMan probe MGI_889 FAM-BHQ (5′-[6-FAM] AGT ACGTACGCAAGTATGAA[BHQ1a-Q]-3′) [27] as described in [2]. qPCR abundance is reported in gene copies per liter of water filtered for collecting microbial biomass under the assumption that DNA extraction had consistent efficiency between samples.

### Metagenome and metatranscriptome processing

Ten metagenomes and eight metatranscriptomes were sequenced via a Joint Genome Institute (JGI) CSP project (Proposal ID 503022) on an NovaSeq S4 (Illumina). Quality-controlled and filtered sequence data were used for MAG generation and transcript recruitment. Metagenomes were both subset and co-assembled to target generation of MAGs for high- and low-abundance microorganisms using the metaWRAP (v1.3.2) pipeline [28] (detailed in the Supplemental Materials & Methods). Metatranscriptomes were analyzed in two ways. First, we used quality-controlled and filtered transcript data (MTF files from JGI Project IDs 1283718–1283727) to obtain transcript abundances only for genes originating in our MAG library. Genes in MAGs were called using Prodigal (v2.6.3), and filtered transcripts were then recruited to these genes using Bowtie2 (v2.4.2). Transcript abundance was calculated using HTSeq [29] with parameter − mode = union. We report the normalized transcript abundance as the transcript count for a given gene divided by the length of the gene and then divided by the transcript abundance in a given sample of two single-copy housekeeping genes related to transcription and DNA replication, namely, DNA-directed RNA polymerase subunit beta (*rpoB*) plus DNA gyrase subunit A (*gyrA*), because these genes are expected to have stable expression. We scaled the abundance of both genes to one across the eight metatranscriptome samples prior to use in normalization. Second, we used the output from JGI annotation projects which use the IMG annotation pipeline (IMGAP v5.0.20) and gene-calling program CRT 1.8.2 on JGI-generated assemblies. We normalized the JGI-generated RNAseq data by dividing the median gene coverage reported for annotated genes on assembly contigs by the sum of the median coverage of *rpoB* plus *gyrA* genes (scaled to one across the eight metatranscriptome samples) in a given sample.

### Phylogenomic and pangenomic analysis

Anvi’o (marie v8) [30] was used for phylogenomic and pangenomic analyses of the AOA and NOB MAGs with detailed description of workflow available in the Supplemental Materials & Methods.

### Statistical analysis

Regressions were made using Pearson correlation with the *cor()* function from base R [31] *stats* package. We used DESeq2 [32] on non-normalized feature counts and removed features with fewer than 50 total counts to test for differential abundance of genes in transcript data from individual MAGs and within bloom versus non-bloom samples. Principal coordinate analysis (PCoA) of MAG abundance was based on Bray–Curtis dissimilarity.

## Results & discussion

### Nitrite and quantitative PCR confirm an ammonia-oxidizing archaea bloom in autumn 2018

**Figure 1:**
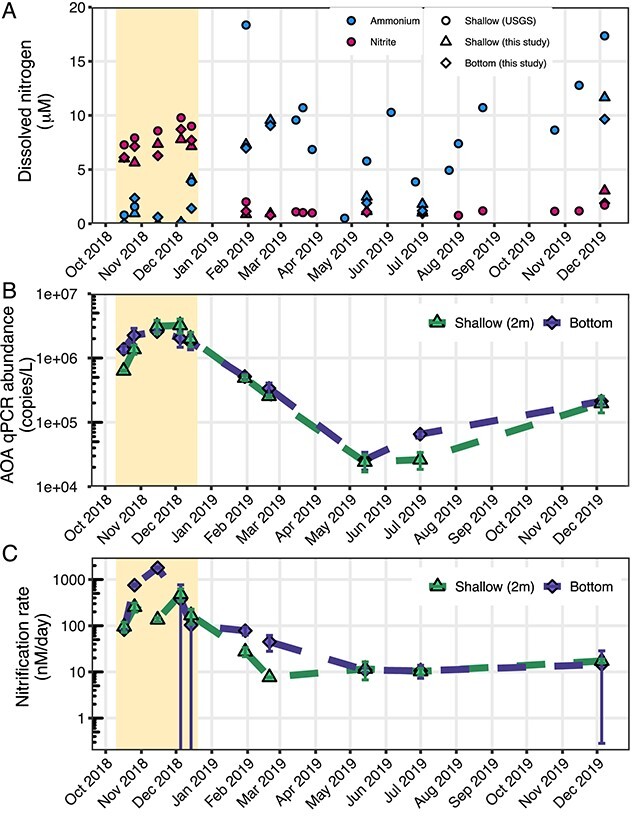
(A) Nitrite and ammonia concentrations measured in this study in both shallow (triangles) and near bottom (diamonds) waters and by USGS for shallow waters (circles). (B) AOA qPCR abundance measured as MGI 16S rRNA gene abundance reported in copies per L with the *y*-axis on a log scale. Error bars indicate the standard deviation between qPCR triplicates. (C) Nitrification rates on a log scale with error bars indicating the standard deviation between triplicate rate measurements. For the two December 2018 samples with standard deviation greater than the mean, lower-bound error bars were truncated to 0.1 so they could be plotted on a logarithmic axis. In (B) and (C), diamonds represent bottom water samples and triangles shallow samples. Highlighted area in figures indicate the AOA bloom samples.

Transient or seasonal nitrite accumulation has been observed in estuaries around the globe and is often caused by the decoupling of ammonia- and nitrite oxidation [33]. The reason for this decoupling varies by system [15–17, 33]; however, blooms of AOA have caused nitrite accumulation in coastal/estuarine systems such as off Sapelo Island [13, 33], in bays of the Yellow Sea [14], and in South SFB [2]. Here, we discuss the results from targeted sampling that took place on 10 cruises from October 2018 to December 2019 in South SFB at USGS Station 27 (Fig. S1). High nitrite concentrations were present in waters from all five cruises in autumn 2018 (mean = 7.0 μM). Elevated nitrite concentrations coincide with low ammonium concentrations, an indication of high ammonia oxidation (Fig. 1). Nitrite concentrations were correlated with MGI Thaumarchaeota 16S rRNA gene abundance (copies per liter) measured via qPCR (referred to as AOA qPCR abundance for the remainder of this text) in both shallow (*R*^2^ = 0.71, *P* value <.001) and bottom waters (*R*^2^ = 0.70, *P* value <.001, Fig. S3). Taken together, these data confirm we sampled during an AOA bloom in 2018. In contrast, nitrite concentrations in autumn of 2019 were not elevated (corresponding to “nonbloom” conditions), which is discussed further below. For the remainder of the manuscript, we categorize the autumn (October through December) 2018 samples as “AOA-bloom samples” and all other samples (February through December 2019) as “nonbloom samples” (Fig. S2).

### High nitrification rates correlated to high ammonia-oxidizing archaea abundance

Estuaries can have a wide range of nitrification rates, ranging many orders of magnitude from < 1 nM day^−1^ to >100 000 nM day^−1^, depending on the ammonia inputs into the estuary [21]. Generally, only estuaries with very high ammonia loading have nitrification rates greater than 1000 nM day^−1^ [21]. Although very few measurements of nitrification rates exist for SFB and focused primarily on North SFB, previous studies found rates ranging from 6.6 to 310 nM day^−1^ in North SFB and ~10 to 200 nM day^−1^ in South SFB [21]. Other studies in the Sacramento River (which flows into North SFB) near large inputs of wastewater effluent have calculated rates in the range of 1500–6400 nM day^−1^ [34, 35]. Here, we report high nitrification rates for South SFB waters, reaching up to 1797 ± 63 nM day^−1^. Rates were generally higher in bottom waters than shallow waters (Fig. 1), in agreement with a previous study [21]. Over the course of the AOA bloom in autumn of 2018, rates in bottom waters increased from 82 ± 1 nM day^−1^ in mid-October to 1797 ± 63 nM day^−1^ in mid-November before declining back down to 103 ± 117 nM day^−1^ in mid-December (Fig. 1). Rates continued to decline after the bloom, reaching a minimum in May 2019 of 11 ± 1 nM day^−1^. In shallow waters, nitrification rates peaked in early December of 2018 at 482 ± 145 nM day^−1^ and reached their lowest in July 2019 at 10 ± 2 nM day^−1^ (Fig. 1). In contrast, phytoplankton biomass was low during the AOA bloom (mean = 2.8 μg L^−1^) and peaked in April 2019 (Fig. S1B) during the annual spring phytoplankton bloom.

Nitrification rates were correlated with AOA qPCR abundance in both shallow (*R*^2^ = 0.73, *P* value <.001) and bottom (*R*^2^ = 0.80, *P* value <.001) waters (Fig. S3) and displayed similar temporal patterns (Fig. 1). The AOA qPCR abundance varied two orders of magnitude between the peak of the AOA bloom and spring and summer, similar to what has been observed near Sapelo Island during a summer AOA bloom [13]. Shallow AOA qPCR abundances were generally lower than bottom water abundances (Fig. 1). In bottom waters, the peak AOA qPCR abundance occurred in November 2018, reaching 2.57E + 06 ± 1.87E + 05 copies L^−1^, and the minimum abundance occurred in May 2019 (2.62E + 04 ± 7.99E + 03 copies L^−1^). Shallow waters had peak AOA qPCR abundance occurring in early December 2018 (3.22E + 06 ± 8.04E + 05 copies L^−1^) and reached the lowest abundance in May 2019 (2.42E + 4 ± 7.27E + 03 copies L^−1^). AOA are enriched in bottom waters of other estuaries [36], perhaps due to their sensitivity to light [37] or competition with phytoplankton. Despite variation in nitrification rates and AOA qPCR abundances in shallow versus bottom waters, nitrite concentrations were generally similar in shallow and bottom waters (Fig. 1). In December 2019 (nonbloom autumn), AOA qPCR abundance was 2.13E + 05 ± 2.15E + 04 copies L^−1^ in bottom waters, fairly similar to the abundance measured in February 2019 (3.38E + 05 ± 27.02E + 04 copies L^−1^). A substantial AOA bloom did not occur in autumn 2019, and our findings support that in nonbloom years, autumn and winter can have comparable AOA abundances and nitrification rates that are higher than those in spring and summer (Fig. 1).

The only other reported measured nitrification rates from South SFB may have also captured an AOA bloom in December 2011, with rates at USGS Station 30 (Fig. S1A) reaching ~200 nM day^−1^ in bottom waters (and ~40 nM day^−1^ in shallow waters) and nitrite concentrations >7 μM at both depths [21]. These rates and nitrite concentrations align well with the values we report for December 2018 shallow (167 ± 60 nM day^−1^ to 482 ± 145 nM day^−1^) and bottom (103 ± 117 nM day^−1^ to 372 ± 393 nM day^−1^) waters at neighboring Station 27. The peak bloom rates in bottom waters at Station 27 (1797 ± 63 nM day^−1^) are in the range of those calculated in the Sacramento River near large inputs of ammonia-rich wastewater effluent (1500–6400 nM day^−1^) [34, 35].

### Ammonia concentrations are high in autumn 2019 when nitrification rates are low

**Figure 2:**
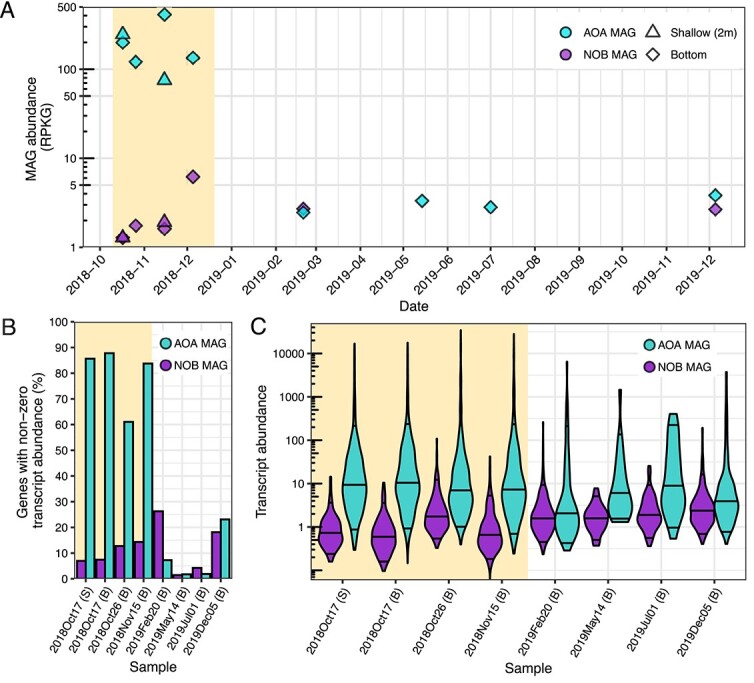
(A) Top panels show the relative abundance in RPKG for the AOA and NOB MAGs over time. (B) Bottom-left panel shows the percentage of genes identified using prodigal in the AOA and NOB MAG that have nonzero transcript abundance at a given time point. (C) Bottom-right panel shows transcript abundance for all genes with nonzero transcript abundance in the AOA and NOB MAGs. Violin plots are a constant width with the 50%, 5%, and 95% quantiles indicated by horizontal lines. Violins are trimmed to the range of data. Highlighted areas indicate the AOA bloom samples in all three panels.

In December 2019, we observe high ammonia and low nitrite concentrations along with lower AOA abundances, AOA transcript abundance, and nitrification rates when compared to December 2018 (Fig. 1). Although AOA are less abundant in qPCR and metagenome data in December 2019 (nonbloom) versus December 2018 (bloom), transcript abundance data indicate that AOA are not totally inactive and are still the dominant nitrifiers (Fig. 2). We also observe similar abundance as in February 2019 in qPCR data (Fig. 1). Determining what conditions determine whether AOA form a massive bloom or not is the subject of further study.

### Nitrosomarinus-like lineage dominates 2018 ammonia-oxidizing archaea bloom with high transcript abundance

To better understand the activity and identity of the blooming AOA lineage, we analyzed 10 metagenomes (6 bloom and 4 nonbloom) and 8 metatranscriptomes (4 bloom and 4 nonbloom) (Fig. S2). Given the higher nitrification rates and AOA qPCR abundances in bottom versus shallow waters, we primarily sequenced bottom water samples. Our multipronged binning efforts yielded 2859 MAGs that were dereplicated at 98% average nucleotide identity (ANI), yielding 292 representative MAGs. This MAG library recruited 10.9%–24.8% of metagenome reads (Table S2). We recovered a total of 71 AOA MAGs (all classified as *Ca.* Nitrosomarinus catalina) originating from bloom samples with a minimum ANI of 99%, which yielded one representative MAG after dereplication: SFB_27S_18Oct17_05_ms_bin_1_strict. This representative AOA MAG has 1545 genes, a genome size of 1.21 Mb, and 97.1% completeness and 0% contamination (Table 1). The *Ca.* Nitrosomarinus catalina MAG recovered from the 2018 AOA bloom samples is highly similar to the MAG generated from samples collected 5 years prior during the 2013 AOA bloom [2], with an ANI of 99.3% and 100% identical *amoA* sequences at the nucleotide level. The two bloom AOA MAGs from SFB are of similar size and quality, with the 2013 AOA MAG containing 30 unique genes and the 2018 AOA MAG containing 38 unique genes when compared with each other and the *Ca.* Nitrosomarinus catalina SPOT01 strain (Fig. S4). Most of these singleton genes were unannotated/of unknown function, with a handful of genes related to nucleotide or amino acid biosynthesis and general cellular maintenance.

**Table 1:** Summary statistics for MAGs of interest.

**Genes w/ significant abundance change**	0	691	25
**Genes w/ nonzero transcript abundance**	1337 (47%)	1447 (94%)	1106 (80%)
**Genes**	2872	1545	1391
**Size (Gb)**	2.5	1.21	1.57
**Contamination (%)**	2.35	0	0.06
**Completeness (%)**	80.8	97.1	84.7
**Genus (GTDB-tk)**	SZUA-226	Nitrosopumilus	MGIIb-O2
**Family (GTDB-tk)**	Nitrospinaceae	Nitrosopumilaceae	Thalassarchaeaceae
**MAG**	SFB_27D_18Dec05_100_mh_bin_22_MF	SFB_27S_18Oct17_05_ms_bin_1_strict	SFB_27S_18Oct17_50_mh_bin_25_strict

**Figure 3:**
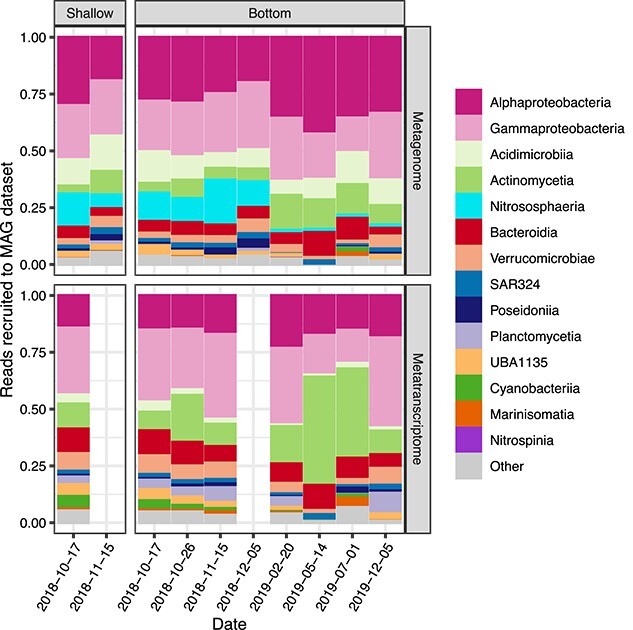
(A) The relative proportion of metagenome reads recruited to the MAG library based on RPKG abundance, highlighting specific MAGs of interest and the 12 most abundant classes outside of that. The MAG library recruited an average of 18.6% of metagenome reads. (B) The relative proportion of transcript reads recruited to the MAG library. The MAG library recruited an average of 13.8% of transcript reads.

**Figure 4:**
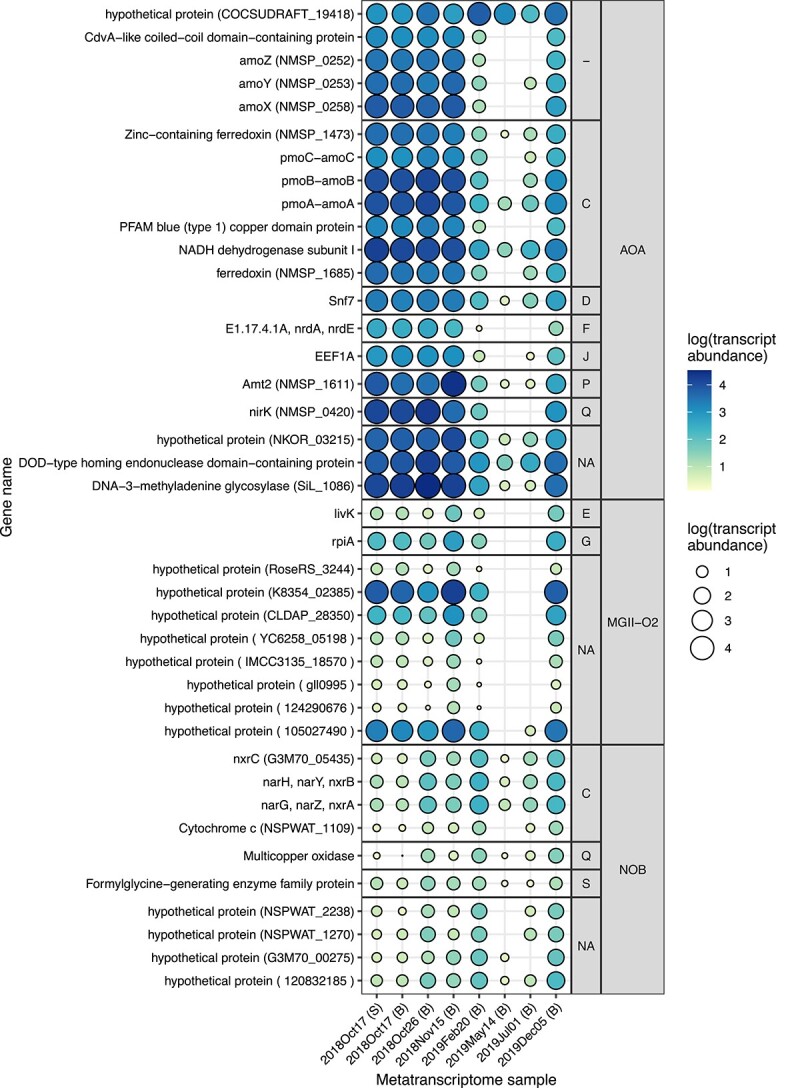
The 20 genes with highest transcript abundance from the representative AOA MAG and top 10 genes with highest transcript abundance for MGII-O2 and NOB MAGs. Point size and color corresponds to transcript abundance on a log scale. UniprotKB protein seed is in parentheses for select proteins and unannotated/unknown function proteins when available. Genes are grouped by COG functional category, including: energy production and conversion (C); cell cycle control, cell division, and chromosome partitioning (D); amino acid metabolism and transport (E); nucleotide metabolism and transport (F); carbohydrate metabolism and transport (G); translation, ribosomal structure, and biogenesis (J); inorganic ion transport and metabolism (P); secondary metabolites biosynthesis, transport, and catabolism (Q); function unknown (S); and unassigned functions (− or NA).

The representative 2018 AOA MAG reached the highest abundance of any single MAG in the dataset (Fig. 3), reaching over 400 reads per kilobase of genome per gigabase of metagenome (RPKG; 1800× coverage) during the bloom (Fig. 2). Bloom samples have very high population and consensus ANI (calculated via inStrain) to the representative AOA MAG, highlighting the low allelic diversity of the dominant AOA lineage during the bloom (Fig. S4). Transcription of genes from this abundant AOA lineage was very high in all four bloom samples (Fig. 2) and 2%–4% of all transcripts (30%–40% of mapped transcripts) recruited to this MAG during the bloom (Fig. 3). A high proportion of genes from the AOA MAG were transcribed, with 1447 (94%) genes having nonzero transcript counts (Fig. 1; Table 1). Transcription of most predicted genes was also observed during the Sapelo Island AOA bloom [13] and translation of the genome (based on proteomics) is high in other marine AOA [38, 39], supporting a general streamlining and nonredundancy of marine AOA genomes. There is also some support for constitutive gene expression by AOA in estuarine environments [4]. The five most highly transcribed genes during the South SFB AOA bloom include: *amoA*, *amoB*, *nirK*, a putative DNA-3-methyladenine glycosylase (SiL_1086), and a high-affinity ammonium transporter (Amt2) (Fig. 4). These findings are in line with previous studies on the activity of estuarine/marine AOA that have found genes related to energy production (particularly *amoA*, *amoB*, *amoC*, and *nirK*), carbon fixation, cell surface processes, and molecular processing to be highly transcribed and translated [13, 38–40]. Other highly transcribed genes in our dataset include *amoC*, superoxide dismutase (SOD2), ferredoxin, an elongation factor, a blue (type 1) copper protein, and several hypothetical proteins that may be part of ammonia oxidation machinery referred to as “amoX,” “amoY,” and “amoZ” [41, 42] (Fig. S5).

AOA could be experiencing oxidative stress and DNA damage from the high rate of their metabolism during the bloom, given the high expression of SOD2 and DNA-3-methyladenine glycosylase—an enzyme involved in DNA base repair, recognizing base lesions from alkylated and deaminated purines [43], and possibly initiating the base excision repair (BER) pathway in archaea [44]. Reactive oxygen/nitrogen species (ROS/RNS) can be produced by aerobic metabolisms, and ammonia oxidation is known to produce nitric oxide (NO) as an intermediate [45–47] making oxidative stress during blooms likely. In line with these findings, the high transcript abundance of SOD2 and DNA repair genes was also observed in the bloom off Sapelo Island [13, 48] and in low-oxygen waters of the Bohai Sea [49].

There are two Amt-like transporters transcribed by the AOA lineage: a low-affinity ammonium transporter (Amt1) that had low expression and decreased over the course of the bloom, as well as a high-affinity ammonium transporter (Amt2) that was the mostly highly expressed gene by AOA and had expression that increased almost an order of magnitude over the bloom (Fig. S5 and S6). The differential expression of Amt1 and Amt2 is likely caused by the low concentrations of ammonia and high ammonia-oxidation rates occurring during the bloom. Culture-based experiments of *Nitrosopumilus maritimus* SCM1 have also observed several-fold higher expression of Amt2 than Amt1 and a decrease in Amt1 expression under low ammonia conditions [50]. Contrastingly, the expression of Amt1 was higher than Amt2 in cultures of *Nitrosopelagicus brevis*; though both genes remained generally highly expressed across growth conditions, they did have relative decreases between the exponential and stationary phase [40].

Alternative sources of ammonia could become increasingly important for AOA as the bloom progresses. Indeed, urea or cyanate can fuel ammonia oxidation in certain environments [51–55]. These alternate sources of ammonia can be used directly by AOA, as evidenced by the high expression of urea transporters by *Nitrosopelagicus brevis* populations in ocean waters [40] or possibly through cross-feeding of ammonia derived from urea or cyanate by NOB to AOA [53, 56–58]. We also previously observed that most *Nitrosomarinus*-like genomes have urease, indicating urea could be important for this lineage [2]. Despite high rates of nitrification and low ammonia concentrations during the AOA bloom in SFB, urease and urea transporters were transcribed at lower levels than both Amt2 and Amt1 by the AOA lineage during the bloom (Fig. S5). Nitrilase (NIT1) also had low expression (Fig. S5). We did not observe signs of ammonia starvation or copper stress from AOA, such as increasing expression of *amoC*, cobalamin synthesis [38], or *hsp20*, or significantly decreasing *amoA* and *amoB* expression [40] (Figs 4, S5, and S6). However, we observe that Amt2 expression increases substantially in November, which could indicate the beginning of ammonia stress.

RNAseq data generated by JGI, which would include genes from organisms for which we did not generate MAGs, showed similar patterns of gene expression related to archaeal ammonia oxidation (Figs 3 and S7). For example, AOA Amt expression increased from mid-October to November whereas *nirK* expression decreased (Fig. S7). The only major difference observed was an increase in archaeal *amoB* transcript abundance over the course of the AOA bloom and a decrease in *amoA,* as opposed to stable expression of these genes for the AOA MAG (Fig. S7). These differences could be related to recruitment of reads to genes from MAGs versus genes from unbinned assembly scaffolds.

### Putative nitrite-oxidizing bacteria metagenome-assembled genome has low abundance and low transcript abundance

From our MAG library, we recovered six MAGs falling within the *Nitrospinaceae* family. After dereplication at 98% ANI, the selected representative *Nitrospinaceae* MAG lacked the key functional genes for nitrite oxidation, namely, nitrite oxidoreductase (NXR) genes. However, we did recover NXR genes from three of the other *Nitrospinaceae* MAGs, so the most complete of these (SFB_27D_18Dec05_100_mh_bin_22_MF) was used as the representative MAG in our analyses. The putative NOB MAG is 2.5 Mb, has 80.8% completeness and 2.4% contamination (Table 1) and falls into the genus SZUA-226 (*Nitrospinaceae*). The other MAGs in this genus found in the Genome Taxonomy Database (GTDB) come predominantly from deep marine sediments, including near the Mariana Trench, sulfidic sediments near a hydrothermal vent in the Guaymas Basin, ferromanganese nodule fields, or cold seeps. In a concatenated ribosomal tree and output from GTDB, the putative NOB MAG is most closely related to genomes from an Oslofjord subsea tunnel biofilm and Guaymas Basin sediments (Fig. S8). Even though the SFB MAG was generated from oxic water column samples, related lineages are from deep sea marine sediments.

The putative NOB MAG was most abundant in the 5 December 2018 metagenome (Fig. 2A). Unfortunately, transcriptome sequencing of the December 2018 samples was unsuccessful. The genes with highest transcript abundance for this NOB MAG were *nxrB* followed by *nxrA*, both of which have peak transcript abundance in February 2019 (Fig. 4). The *nxrB* for the putative NOB MAG is related to sequences from marine sediments and a coral (Fig. S8). A gene annotated as the nitrite oxidoreductase gamma subunit (*nxrC*) found adjacent to *nxrB and nxrA* also had high transcript abundance (Fig. 4). Other genes with high transcript abundance were related to energy generation and cellular processes (Fig. 4). The gene *pilA* had high transcript abundance, and many other genes related to pilus and flagella synthesis and chemotaxis were expressed, indicating possible motility of this lineage (Fig. S8). This putative NOB MAG did not have ectoine synthesis genes reported previously for *Nitrospinaceae* isolates [59] or glycine betaine ABC transporter (OpuABD) genes but did contain spermidine transport system (*potA*) and synthase (*speE*) genes, a small conductance mechanosensitive channel (*mscS*), and transcriptional regulatory protein *ompR* that could be used for dealing with osmotic stress, an important function in an estuary with fluctuating salinity. Although cobalamin synthesis genes were lacking, the MAG did encode genes for a cobalamin transporter, with one (*btuF)* having nonzero transcript abundance in February 2019, indicating these NOB could scavenge cobalamin [60].

In the ocean, AOA and NOB can co-occur and have cross-feeding interactions based on their linked metabolisms [56, 61]. AOA and NOB can also compete for N sources for assimilatory purposes, leading to a diverging uptake/preferences for N compounds [57, 62]. Despite potentially strong competition for ammonia in South SFB, the NOB MAG in this study did not encode genes for urease or cyanase. Several *Nitrospinaceae* lack canonical mechanisms for dealing with reactive oxygen species such as SOD or catalase and may depend on other microorganisms to deal with oxidative stress [59, 62]. In contrast, SOD and other ROS defense genes have recently been recovered from some *Nitrospina* genomes [63, 64]. The NOB MAG contained genes annotated as a copper/zinc SOD (*sodC*) and antioxidant enzymes *ahpC* and *Tpx*, however, were all expressed at low levels (Fig. S8). Given the contrasting transcript abundance for oxidative stress response genes from AOA and NOB, perhaps oxidative stress could play a role in the decoupled growth between nitrite and ammonia oxidizers during the bloom.

The putative NOB MAG had low and somewhat-stable transcript abundance during the AOA bloom with an increased number of genes transcribed in February and December 2019 (Fig. 2). Like the AOA lineage, the NOB lineage also had the lowest gene expression in May and July. Although we do not see evidence for increased NOB activity over the course of the AOA bloom, we are lacking transcript data for the second month of the bloom (December 2018) when NOB showed an increase in relative abundance in metagenomic data (Fig. 2). Given the persistence of high nitrite concentrations through December of 2018, it appears unlikely that NOB activity meaningfully increased during this time when we lack data. In JGI-generated RNAseq data, which includes assemblies that were not binned, we also see the highest transcript abundance for *nxrB* occurring in February and December 2019 from scaffolds predominantly classified as coming from *Nitrospina* (Fig. S7). Winter conditions (higher turbidity, lower salinity, and colder temperatures) appear more favorable for NOB in this system, despite the large amount of available nitrite occurring during the AOA bloom in autumn. Further culture-based work is necessary to assess if the dominant NOB and AOA strains identified in South SFB have different salinity optima, temperature optima, or maximum growth rates that could contribute to the decoupling of the two nitrifiers during the AOA bloom. Additionally, SFB receives high levels of wastewater discharge and urban runoff that input many contaminants of concern, including but not limited to per- and polyfluoroalkyl substances (PFASs; commonly referred to as “forever chemicals”), microplastics, and quaternary ammonia compounds (QACs; surfactants commonly used in products as antimicrobials), that could be inimical to NOB. PFASs can inhibit nitrification in wastewater [65], constructed wetlands [66, 67], and soils [68], where ammonia-oxidizing bacteria (AOB) may be more strongly impacted than AOA [69]. PFASs have been widely found in SFB waters, sediments, and biota [70, 71]. Microplastics are ubiquitous in SFB [72, 73], and studies have found that certain microplastics inhibit nitrification [74, 75], with some particularly inhibiting nitrite oxidation more than ammonia oxidation [76], as well as inhibit QAC breakdown [75]. QACs have also been shown to inhibit nitrifiers, particularly AOB and NOB, in culture [77], wastewater [78, 79], and natural aquatic environments [80]. QACs have increased in use since the COVID-19 pandemic and a suite of QACs, though predominantly those used in disinfectant products, have been observed in wastewater effluent and stormwater runoff into SFB as well as in bay surface waters and sediments [81]. Additional studies of these contaminants in the natural environment and their impacts on the growth of the specific dominant nitrifier strains in South SFB could yield insight into the decoupled activity of the two nitrifier guilds during AOA blooms.

We did not recover an AOB MAG, and previous findings support a low abundance of AOB in South SFB [2]. However, in JGI-generated RNAseq data, we see some increased transcript abundance of ammonia-oxidation genes from AOB during February 2019 (Fig. S7). AMO genes from AOB still have far lower transcript abundance than AOA AMO genes in February (Fig. S7). We also see that putative bacterial NXR and AMO genes are most highly transcribed in December of 2019 when we do not see a large bloom of AOA, though the AOA still have the highest transcript abundance of the nitrifiers at this time. The lower transcript abundance for NOB and AOB compared to AOA during the bloom is similar to patterns observed for a summer AOA bloom off Sapelo Island [13].

### Potential impacts of ammonia-oxidizing archaea bloom on the microbial community difficult to identify

We generated MAGs for 292 microbial lineages from over 18 phyla after dereplication at 98% ANI. The highest number of MAGs were generated from the *Proteobacteria*, *Actinobacteriota*, and *Bacteroidota*. Classes within these phyla, such as *Alphaproteobacteria*, *Gammaproteobacteria*, *Acidimicrobiia*, and *Actinomycetia*, had high relative and transcript abundance (Fig. 3). In addition to the AOA MAG, several MAGs from *Pelagibacter*, an *Actinomarina,* and a *Planktomarina termperata* had high relative abundance in line with their predominance in 16S rRNA gene amplicon libraries from this station in 2012–2014 [3]. Identifying potential interactions between the blooming AOA and other microbial lineages based on co-occurrence patterns of MAGs or differential transcript abundance between bloom and nonbloom samples was difficult. Our analyses seemingly only identified MAGs with similar seasonal distribution as AOA and not necessarily microorganisms that were responding to the bloom conditions such as low ammonia or high ROS/RNS (Figs S9 and S10; see Supplemental Results and Discussion for more details). A MG IIb-O2 *Euryarchaea* (*Ca.* Poseidonales) MAG had high relative abundance and transcript abundance during the AOA bloom and reached peak relative abundance in November along with the AOA MAG (Figs 2 and S11). Although it has been proposed that MGIIb could break down organic matter and release ammonia through ammonification leading to a coinciding bloom of AOA in the Yellow Sea [14], there was no clear interaction between the MGIIb-O2 and *Ca.* Nitrosomarinus catalina lineage during the bloom based on the gene transcript abundance data. Genes from the MGIIb MAG with high transcript abundance were annotated as hypothetical or associated with cellular processes and not degradation of organic matter (Fig. S11; See Supplemental Results and Discussion for more details), making it unclear whether these two archaea are dependent on one another for reaching high abundance or if their peak abundance is related primarily to seasonal conditions.

## Conclusions

We report high nitrification rates for South SFB and describe the microbial dynamics of an AOA bloom responsible for those biogeochemical rates. We find that AOA abundance increases orders of magnitude between the bloom and nonbloom seasons in both qPCR and metagenomic data. We also find that a *Ca.* Nitrosomarinus catalina-like lineage is highly active during the bloom whereas a putative NOB within the *Nitrospinaceae* has orders of magnitude lower transcript abundance. The abundance and transcript abundance of AOA and NOB appear to be decoupled during the bloom, allowing nitrite to accumulate in the oxic water column. The high levels of ammonia oxidation may impact other members of the microbial community, possibly responsible for the high transcript abundance of ammonia transporters in some lineages and of genes associated with using alternate N sources or coping with oxidative stress. We also identify an MGIIb MAG that has high abundance and activity during the AOA bloom. However, the interaction between the two archaea lineages is unclear based on transcriptional data, and their high abundances could simply be related to similar ecophysiological properties. We also support findings that AOA are generally more active in bottom waters of estuaries and coasts than in surface waters, likely related to light inhibition or competition with phytoplankton for ammonia. Although high N loading in SFB does not yet fuel regular harmful algae blooms, our study shows that high ammonia concentrations currently support active AOA blooms. The decoupling of AOA and NOB activity and abundance could highlight different growth rates between the dominant nitrifier strains or conditions inimical to NOB in SFB despite the high levels of nitrite and oxygen available in the water column during the AOA bloom. This study highlights just some of the current impacts of high nutrient inputs and a need to further study the potential impacts of micropollutants, warmer temperatures, and saltier waters (due to decreased freshwater inputs and more evaporation) on microbial N-cycling processes, particularly ammonia and nitrite oxidation.

## Supplementary Material

Supplementary_information_15Jul24_clean_wrae148

## Data Availability

Metatranscriptomes are available under NCBI Bioprojects PRJNA709957 through PRJNA709964. Raw metagenomes are available in NCBI in PRJNA1046415 through PRJNA1046424. Quality controlled and filtered metagenomes are available from the JGI Genome Portal under accession no. 3300044246, 3300044491, 3300044512, 3300044513, 3300044657, 3300044670, 3300044674, 3300044676, 3300044723, and 3300044724. MAGs from 2018 to 2019 are deposited in Bioproject no. PRJNA819089. The 2013 AOA MAG is available on NCBI under accession JAJETX000000000. SFB water quality data are available from the USGS database [18]: https://sfbay.wr.usgs.gov/water-quality-database/. Additional nutrient data are included in Table S1.
