# Supplementary material for: Dynamics and activity of an ammonia-oxidizing archaea bloom in South San Francisco Bay
Source: ISME J. 2024 Jul 30;18(1):wrae148. doi: 10.1093/ismejo/wrae148 (PMC11334935; doi:10.1093/ismejo/wrae148)
Supplement: Supplementary_information_15Jul24_clean_wrae148 [file supplementary_information_15jul24_clean_wrae148.docx]

Supplementary Information for

Dynamics and activity of an ammonia-oxidizing archaea bloom in South San Francisco Bay

Anna Rasmussen^1^ and Christopher A. Francis^1,2^*

^1^Department of Earth System Science and ^1^Oceans, Stanford University, Stanford, CA 94305, USA

**Supplemental Methods**

*Sampling*

We sampled on October 17^th^, October 26^th^, November 15^th^, December 5^th^, and December 14^th^ in 2018 and seasonally on February 20^th^, May 14^th^, July 1^st^, and December 5^th^ in 2019.

*Stable Isotope Incubations*

Stable isotope incubations were set up in triplicate in 1-gallon gas sampling multi-layer foil Restek bags with combo valve and septum port (Restek Corporation, Bellefonte, PA, part #22950) after set up described in [1] filled with 450 mL of 80 μm prefiltered bay water. ^15^N-ammonium (99 atom% ^15^N) was added to the Restek bags targeting ~10% ambient ammonia concentrations (from 0.05 to 0.3 μM during bloom versus non-bloom samples). Stable isotope incubations were stored in a large cooler filled with Bay water. After initial (T0) addition of ^15^N-ammonium and after 6 hours (T6) of incubation, 60mL of water was subsampled and filtered through 0.22 μm pore-sized filters before storage at -20°C. ^15^N-NO_x_ in both T_0_ and T_6_ subsamples was measured by the University of California Davis Stable Isotope Facility (Davis, CA) using the denitrifier method [2] to convert ^15^N-NO_x_ to ^15^N-N_2_O, which was then measured via isotope-ratio mass spectrometry (<https://stableisotopefacility.ucdavis.edu/nitrate-no3-water>). Based on atom fraction, rates were then calculated using an endpoint model to estimate flux from the ammonium to NO_x_ (nitrate + nitrite) pool as described in [3] using the following equations:

Equation 1:

$${Rate}_{nit} = \frac{(\left[ {NO}_{x} \right])\left( {AF}_{{15N-NOx}_{f}} - {AF}_{{15N-NOx}_{i}} \right)}{\left( {AF}_{15N-{NH}_{4_{sp}}^{+}} \right)x t}$$

Equation 2:

$${AF}_{15N-{NH}_{4_{sp}}^{+}}= \frac{(\left[ {NH}_{4_{i}}^{+} \right])({AF}_{15N-{NH}_{4_{i}}^{+}}) + [{NH}_{4_{add}}^{+}]({AF}_{15N-{NH}_{4_{add}}^{+}})}{[{NH}_{4_{i}}^{+}]+[{NH}_{4_{add}}^{+}]}$$

Where the rate of nitrification (${Rate}_{nit}$) is equal to the concentration of NO_x_ times the atom fraction of the final NO_x_ pool (${AF}_{{15N-NOx}_{f}}$) minus the initial NO_x_ pool (${AF}_{{15N-NOx}_{i}}$). This is divided by the atom fraction of the ammonium pool after spiking with ^15^N-NH_4_^+^, which is described in equation 2.The ${AF}_{15N-{NH}_{4_{sp}}^{+}}$ is equal to the initial ammonium concentration (${NH}_{4_{i}}^{+}$) times the standard atom fraction of ^15^N-NH_4_^+^ (${AF}_{15N-{NH}_{4_{i}}^{+}}$equal to 0.003663) plus the concentration of ^15^N-NH_4_^+^ $({NH}_{4_{add}}^{+}$ ) added times the enriched atom fraction (${AF}_{15N-{NH}_{4_{add}}^{+}}$equal to 0.99) divided by the initial plus added ammonium concentrations. All NO_x_ and ammonia concentrations used in calculations were measured in this study. Generally, nutrient concentrations measured by USGS in shallow waters were similar but slightly higher than those measured in this study (see Fig. 1). If ammonia was undetectable, we used a value of 0 to help give a conservative estimate of nitrification rates by assuming the only ammonia in the sample was the ^15^N-NH_4_^+^ we added. Rates are reported as nanomoles of ammonia-N converted to NOx-N per L per day (nM day^-1^).

*DNA extraction*

Both DNA and RNA was extracted using a modified phenol co-extraction method [4, 5]. Briefly, frozen filters were crushed in sterile Whirl-Pak® bags (Nasco Sampling, Pleasant Prairie, WI) and filter fragments were added to a sterile microcentrifuge tube containing a hexadecyltrimethylammonium bromide (CTAB) and polyvinyl pyrrolidone (PVP) solution as well as 3 sizes (0.1, 0.5, and 5 mm) of sterile glass beads. Filters were then homogenized using a FastPrep bead beater (MP Biomedicals, Santa Ana, CA) at speed 5.5 for 30s. Supernatant was mixed with neutral phenol:chloroform:isoamyl alcohol 3 times to remove proteins. Nucleic acids were precipitated with cold ethanol, washed 3 times with ethanol, and then eluted into a 100 μL of nuclease-free water and split into two aliquots, one for RNase and one for DNase treatment using the DNase I kit (Life Technologies) and RNase I kit (Life Technologies) and following manufacturer’s instructions. After RNase and DNase treatment, we performed a final phenol:chloroform (pH 8 for DNA, pH 5 for RNA) clean-up and 2 ethanol wash steps. Final DNA and RNA pellets were resuspended in 50 μL of 55°C and 95°C, respectively, sterile nuclease-free water. DNA and RNA was quantified using the Qubit dsDNA Broad Range assay (Life Technologies, Grand Island, NY) and stored at -80°C.

*Metagenome sequencing, assembly, and binning*

Ten metagenomes were sequenced via a Joint Genome Institute (JGI) CSP project (Proposal ID 503022) on an NovaSeq S4 (Illumina). Quality Controlled Filtered Raw metagenome data (JGI Project Ids 1283708 to 1283717) was downloaded from JGI Genome Portal for assembly, binning, and refining using the metaWRAP (v1.3.2) pipeline [6]. Metagenomes were assembled using MEGAHIT (v1.1.3) [7] following default parameters. Metagenomes were subset using seqtk (seed -s100) at 1%, 3%, 5%, 10%, 20%, and 50% and co-assembled to allow for targeted assembly of high and low abundance nitrifiers. Additional assemblies were made using metaSPAdes (v3.13.0) [8] for 1% through 10% subsets of metagenome reads. Single sample assemblies were binned using contigs > 2000 nt with two methods. The first used both MetaBAT2 (v2.12.1) [9] and MaxBin 2.0 (v2.2.6) [10] using fastq files only from the assembly sample of origin. These bins were consolidated and filtered using *metawrap bin_refinement* to be > 50% completeness and have < 10% contamination as calculated via CheckM (v1.1.3) [11] and then reassembled with metaSPAdes using strict or permissive algorithm using *metawrap reassemble_bins*. The second method aimed to improve coverage information by mapping multiple fastq files to assemblies for binning with MetaBAT2, MaxBin 2.0, and CONCOCT (v1.1.0) [12] followed by *metawrap_bin_refinement*. Co-assemblies were binned using contigs > 2500nt using MetaBAT2, MaxBin 2.0, and CONCOCT and multiple fastq files followed by *metawrap_bin_refinement*. Lastly, all of the MAGs were consolidated and dereplicated using dRep (v2.3.2) [13] at 98% ANI. Taxonomic classification for MAGs was made using the Genome Taxonomy Database toolkit (GTDB-tk) [14] with the database release RS220. Reads were competitively recruited to the dereplicated MAG library using Bowtie2 (v2.4.2) [15] and the default parameters. Abundances are displayed as unpaired reads recruited per genome size of MAG in kilobases of MAG divided by gigabase of metagenome (RPKG) and coverage values calculated using inStrain [16]. Genes were called using Prodigal (v2.6.3) [17] and translated gene annotations were performed using GhostKOALA [18]. For select MAGs of interest, translated sequences were also annotated using eggNOG-mapper (v 2.1.9) [19].

*Phylogenomic and pangenomic analysis*

The AOA MAG was compared with our previously generated SFB AOA bloom MAG [20] and *Ca.* Nitrosomarinus catalina SPOT01. Genomes for the GTDB species representatives in the *Nitrospinales* family (n = 74) in GTDB (RS220) were downloaded from NCBI to compare to our putative NOB MAG. For all pangenomic analyses, contigs shorter than 500bp in length were excluded from the analysis. Conserved ribosomal and housekeeping genes were annotated using *anvi-run-hmms* with the argument *–also-scan-trnas* which uses tRNAscan-SE (v2.0.12). All genomes were annotated using COG, Kegg KO, and pfams through the anvi’o pipeline with Diamond set to fast. Gene sequences of interest (*amoA*, *nxrB*) were extracted using *anvi-get-sequences-for-gene-clusters*. The ANI of genomes was calculated using pyANI [21] through anvi’o using default parameters. For *Nitrospinaceae*, concatenated and amino acid sequences aligned using MUSCLE [22] were retrieved from MAGs using *anvi-get-sequences-for-hmm-hits* for all genomes containing 27 out of 38 ribosomal genes, leaving 66 genomes in the analysis. Genes not occurring in at least 62 of 66 genomes were removed yielding 21 remaining ribosomal genes. Trimal was used to remove gaps in the 3,205 length amino acid alignment with less than 50% coverage. Then IQ-TREE -m MFP was used for extended model selection for phylogeny of NOB genomes. IQ-TREE was used to construct the final phylogenomic tree using model LG+R6 with 1000 bootstraps. Nucleotide sequences of genes annotated as *narG*/*nxrB* were aligned using MAFFT and the tree was made using PhyML with100 bootstraps.

*Statistical analyses*

The SPIEC-EASI network was made using *spiec.easi* in R with the following parameters: method = “mb”, lambda.min.ratio = 1e-2, nlamda = 20, pulsar.params = (list(rep.num=50)).

**Supplemental Results and Discussion**

*AOA ammonia oxidation transcript abundance ratios over time*

Due to low transcript abundance by AOA in non-bloom samples, we could not robustly identify seasonal variations in expression of ammonia-oxidation genes. We examined the ratio of ammonia-oxidation and transport genes to *amoA* transcripts to understand transcript abundance patterns over the course of the bloom. For example, the *nirK*:*amoA* transcript abundance ratio decreases over the course of the bloom whereas the Amt2:*amoA* ratio increases over the course of the bloom. The *amoC*:*amoA*, and *amoB*:*amoA* ratios remain relatively stable over the bloom (Fig. S6). During the AOA bloom off Sapelo Island, the reported ratios of *amoC*:*amoA* and *nirK*:*amoA* were stable across seasons whereas *amoB*:*amoA* varied by season [23].

*AOA bloom impacts N supply and oxidative stress for other microbes*

Given the high rates of ammonia oxidation, we also looked at general patterns in genes related to N-uptake from all non-AOA and non-NOB MAGs during the bloom. An Amt-like ammonia transporter had high transcript abundance in all samples and reached peak expression in May 2019 (Fig. 5) during the annual spring phytoplankton bloom [24, 25] (seen in chlorophyll *a* data in Fig. S1). Genes related to urea transport and utilization had highest transcript abundance during the AOA bloom and in May. One nitrate/nitrite transporter also has peak transcript abundance during the AOA bloom. Although generally expressed at low levels, nitrile hydratase and cyanate lyase had highest expression in November 2018 (Fig. 5). In summary, transcription of genes for ammonia transporters, urea transport and utilization, nitrile hydratase, and cyanate lyase was typically highest in May 2019 (Fig. S9) during the annual spring phytoplankton bloom [23,24] or during the AOA bloom.

Given the potential for oxidative stress, we also assessed microbial antioxidant systems, including superoxide dismutase, catalase, thioredoxin, and glutathione [26]. Generally, highest transcript abundance of all of these systems occurred in the shallow sample or during the phytoplankton bloom in May, with some genes having higher expression during the AOA bloom. These findings support that high rates of ammonia oxidation could produce similar stress from ROS/RNS as produced by other major sources in marine waters, such as photochemical reactions in surface waters or by photosynthesis [27].

*Potential interactions between AOA and other microbial lineages*

Despite having linked metabolisms, the major AOA and NOB lineages in South SFB appear to have decoupled abundance and activity (Fig. 3). Predicting potential interactions between microbial lineages based on co-occurrence patterns or changes in transcript abundance was difficult. Ten MAGs were significantly associated with the AOA bloom MAG based on a SPIEC-EASI co-occurrence network of MAG abundance. Additionally, differential abundance testing identified 102 of the 292 MAGs with at least one gene with significantly (*Padj* value < 0.05) higher transcript abundance in AOA bloom versus non-bloom samples, though over 30 of these had only one gene with significant differential transcript abundance (Fig. S10). These MAGs generally followed two general patterns, with MAGs either having similar abundance patterns as AOA (peak abundance in fall) or stable abundance throughout the year. A PCoA of all MAGs shows that many of the MAGs with higher numbers (>5 or >10) of genes with differential transcript abundance are more closely associated with AOA bloom samples (Fig. S10A). In general, genes with high transcript abundance from these MAGs with similar temporal abundance patterns as AOA were related to general cellular processes, growth, and metabolism. Similarly, 7 of the 10 MAGs that co-occur with the AOA MAG contained genes with significantly higher transcript abundance during the bloom, but genes were predominantly related to growth and cellular processes and not necessarily related to a response to the bloom. A UBA7326 (*Thalassobaculaceae*) has higher transcript abundance for a urea transport gene, *urtA*, during the bloom that could be related to competition for ammonia. However, the patterns we observe do not necessarily indicate a specific response to the AOA bloom by most of these organisms, but rather suggest these lineages may have some similar ecophysiological properties, leading to increased abundance and activity during the bloom time. There were also MAGs with only a few genes with significant differential transcript abundance between AOA bloom and non-bloom samples. These MAGs tended to have consistent abundance in metagenomes over the course of the entire year or were of low relative abundance in metagenomes (Fig. S10). The genes with significant differences in transcript abundance between bloom and non-bloom samples for these more “generalist” MAGs included many hypothetical proteins, ribosomal proteins, bacterial rhodopsins, and genes related to general metabolism. Thus MAGs that had different seasonal distributions from AOA and significant differences in transcript abundance between bloom and non-bloom samples also did not appear to be expressing genes related to the AOA bloom. A SAR86 MAG in the genus D2472 transcribed more catalase during the AOA bloom than in non-bloom samples and also represented “generalists” with stable abundance in metagenome samples and transcript abundance throughout the year. The transcript abundance patterns of their catalase could be related to biogeochemical impacts of the AOA bloom.

*MG II* Euryarchaea *MAG*

We identified a MG II *Euryarchaea* (*Ca.* Poseidonales) MAG with high abundance and high transcript abundance during the AOA bloom (Figs. 2 & S11). This abundant and active *Ca.* Poseidonales MAG was classified to the species level as MG11b-O2 sp030828405 using GTDB-tk and is related to MAGs from the North Sea, Mediterranean Sea, and off the coast of Spain. Genes from this MG11b-O2 MAG have some of the highest transcript abundances outside of genes from the AOA MAG, which dominate the metatranscriptome. Of the 1106 genes from the MG11b-O2 sp030828405 *Euryarchaea* MAG that had non-zero transcript abundance in our dataset, 21 had significant changes in abundance in bloom vs. non-bloom samples (Table 1). Many of the genes with the highest transcript abundance (11 of top 20) for this MAG were annotated as hypothetical proteins (Fig. S11) and BLAST results yielded only hypothetical proteins from other *Euryarchaea* genomes. In contrast to MGIIa (*Ca.* Posiedonaceae), which generally have higher abundances in summer and are associated with phytoplankton blooms, MGIIb have been shown to have peak abundances generally occurring in fall and winter, driven more by availability of inorganic nutrients [28]. MGIIb (*Ca.* Thalassarchaeaceae [28]) and AOA have been found to co-occur in both the North Sea [29] and Yellow Sea [30]. In the North Sea, MGIIb (and AOA) reached peak abundances prior to annual phytoplankton blooms and encoded more transporters and peptidases than MGIIa [29].

Most of the genes with high transcript abundance for the MGIIb MAG were hypothetical or associated with cellular processes such as transcription, translation, replication, repair, and protein modification (Fig. S11), similar to findings off of Sapelo Island [30]. Although the coinciding blooms of AOA and MGIIb during blooms observed in the Yellow Sea could indicate these *Euryarchaea* are breaking down organic matter and releasing ammonia through ammonification [29], in our dataset we do not see high transcript abundance of degradation genes in the MGIIb MAG making any potential interaction of the MGIIb-O2 and *Ca.* Nitrosomarinus catalina lineage during the bloom unclear. A global analysis found that MGIIb-O2 genus has a peak abundance around 16°C [27], which is the general temperature of South SFB waters in the autumn (Fig. S1). Thus, the environmental conditions during the AOA bloom may also be favorable to MGIIb-O2 and the two archaeal lineages may not necessarily be aiding one another in blooming.

The high abundance of a *Euryarchaea* MAG during the 2018 AOA bloom is in contrast to what we observed in our previous study of an AOA bloom in autumn 2013, where *Euryarchaea* were of low relative abundance in South Bay [21]. Although the difference in *Euryarchaea* abundance between the two datasets could highlight variation in communities over the years, it may also reflect the different size fractions (0.22 to 80 µm versus 10 µm in 2013) and the inclusion of larger or particle-associated microorganisms in 2018 compared to in 2013. Although *Euryarchaea* are found in both free-living and particle-associated fractions, particle attachment or cell aggregation appear to be important in this group [27].

**Supplemental References**

1. Santoro AE, Buchwald C, Knapp AN, Berelson WM, Capone DG, Casciotti KL. Nitrification and Nitrous Oxide Production in the Offshore Waters of the Eastern Tropical South Pacific. *Global Biogeochemical Cycles* 2021; **35**: e2020GB006716.

2. Sigman DM, Casciotti KL, Andreani M, Barford C, Galanter M, Böhlke JK. A Bacterial Method for the Nitrogen Isotopic Analysis of Nitrate in Seawater and Freshwater. *Anal Chem* 2001; **73**: 4145–4153.

3. Damashek J, Casciotti KL, Francis CA. Variable Nitrification Rates Across Environmental Gradients in Turbid, Nutrient-Rich Estuary Waters of San Francisco Bay. *Estuaries and Coasts* 2016; **39**: 1050–1071.

4. Campbell BJ, Kirchman DL. Bacterial diversity, community structure and potential growth rates along an estuarine salinity gradient. *The ISME Journal* 2013; **7**: 210–220.

5. Dempster E l., Pryor K v., Francis D, Young J e., Rogers H j. Rapid DNA Extraction from Ferns for PCR–Based Analyses. *BioTechniques* 1999; **27**: 66–68.

6. Uritskiy GV, DiRuggiero J, Taylor J. MetaWRAP—a flexible pipeline for genome-resolved metagenomic data analysis. *Microbiome* 2018; **6**: 158.

7. Li D, Liu C-M, Luo R, Sadakane K, Lam T-W. MEGAHIT: an ultra-fast single-node solution for large and complex metagenomics assembly via succinct de Bruijn graph. *Bioinformatics* 2015; **31**: 1674–1676.

8. Nurk S, Meleshko D, Korobeynikov A, Pevzner PA. metaSPAdes: a new versatile metagenomic assembler. *Genome Res* 2017; **27**: 824–834.

9. Kang DD, Li F, Kirton E, Thomas A, Egan R, An H, et al. MetaBAT 2: an adaptive binning algorithm for robust and efficient genome reconstruction from metagenome assemblies. *PeerJ* 2019; **7**: e7359.

10. Wu Y-W, Simmons BA, Singer SW. MaxBin 2.0: an automated binning algorithm to recover genomes from multiple metagenomic datasets. *Bioinformatics* 2016; **32**: 605–607.

11. Parks DH, Imelfort M, Skennerton CT, Hugenholtz P, Tyson GW. CheckM: assessing the quality of microbial genomes recovered from isolates, single cells, and metagenomes. *Genome Res* 2015; **25**: 1043–1055.

12. Alneberg J, Bjarnason BS, de Bruijn I, Schirmer M, Quick J, Ijaz UZ, et al. CONCOCT: Clustering cONtigs on COverage and ComposiTion. *arXiv:13124038 [q-bio]* 2013.

13. Olm MR, Brown CT, Brooks B, Banfield JF. dRep: a tool for fast and accurate genomic comparisons that enables improved genome recovery from metagenomes through de-replication. *ISME J* 2017; **11**: 2864–2868.

14. Chaumeil P-A, Mussig AJ, Hugenholtz P, Parks DH. GTDB-Tk: a toolkit to classify genomes with the Genome Taxonomy Database. *Bioinformatics* 2020; **36**: 1925–1927.

15. Langmead B, Salzberg SL. Fast gapped-read alignment with Bowtie 2. *Nat Methods* 2012; **9**: 357–359.

16. Olm MR, Crits-Christoph A, Bouma-Gregson K, Firek BA, Morowitz MJ, Banfield JF. inStrain profiles population microdiversity from metagenomic data and sensitively detects shared microbial strains. *Nat Biotechnol* 2021; **39**: 727–736.

17. Hyatt D, Chen G-L, LoCascio PF, Land ML, Larimer FW, Hauser LJ. Prodigal: prokaryotic gene recognition and translation initiation site identification. *BMC Bioinformatics* 2010; **11**: 119.

18. Kanehisa M, Sato Y, Morishima K. BlastKOALA and GhostKOALA: KEGG Tools for Functional Characterization of Genome and Metagenome Sequences. *J Mol Biol* 2016; **428**: 726–731.

19. Huerta-Cepas J, Forslund K, Coelho LP, Szklarczyk D, Jensen LJ, von Mering C, et al. Fast Genome-Wide Functional Annotation through Orthology Assignment by eggNOG-Mapper. *Molecular Biology and Evolution* 2017; **34**: 2115–2122.

20. Rasmussen AN, Francis CA. Genome-Resolved Metagenomic Insights into Massive Seasonal Ammonia-Oxidizing Archaea Blooms in San Francisco Bay. *mSystems* 2022; **7**: e01270-21.

21. Pritchard L, Glover RH, Humphris S, Elphinstone JG, Toth IK. Genomics and taxonomy in diagnostics for food security: soft-rotting enterobacterial plant pathogens. *Anal Methods* 2015; **8**: 12–24.

22. Edgar RC. MUSCLE: multiple sequence alignment with high accuracy and high throughput. *Nucleic Acids Research* 2004; **32**: 1792–1797.

23. Hollibaugh JT, Gifford S, Sharma S, Bano N, Moran MA. Metatranscriptomic analysis of ammonia-oxidizing organisms in an estuarine bacterioplankton assemblage. *ISME J* 2011; **5**: 866–878.

24. Cloern JE. Phytoplankton bloom dynamics in coastal ecosystems: A review with some general lessons from sustained investigation of San Francisco Bay, California. *Reviews of Geophysics* 1996; **34**: 127–168.

25. Carstensen J, Klais R, Cloern JE. Phytoplankton blooms in estuarine and coastal waters: Seasonal patterns and key species. *Estuarine, Coastal and Shelf Science* 2015; **162**: 98–109.

26. Staerck C, Gastebois A, Vandeputte P, Calenda A, Larcher G, Gillmann L, et al. Microbial antioxidant defense enzymes. *Microbial Pathogenesis* 2017; **110**: 56–65.

27. Zinser ER. The microbial contribution to reactive oxygen species dynamics in marine ecosystems. *Environmental Microbiology Reports* 2018; **10**: 412–427.

28. Rinke C, Rubino F, Messer LF, Youssef N, Parks DH, Chuvochina M, et al. A phylogenomic and ecological analysis of the globally abundant Marine Group II archaea ( Ca . Poseidoniales ord. nov.). *The ISME Journal* 2018; 1.

29. Orellana LH, Ben Francis T, Krüger K, Teeling H, Müller M-C, Fuchs BM, et al. Niche differentiation among annually recurrent coastal Marine Group II Euryarchaeota. *ISME J* 2019; **13**: 3024–3036.

30. Kim J-G, Gwak J-H, Jung M-Y, An S-U, Hyun J-H, Kang S, et al. Distinct temporal dynamics of planktonic archaeal and bacterial assemblages in the bays of the Yellow Sea. *PLOS ONE* 2019; **14**: e0221408.

31. Santoro AE, Dupont CL, Richter RA, Craig MT, Carini P, McIlvin MR, et al. Genomic and proteomic characterization of “Candidatus Nitrosopelagicus brevis”: An ammonia-oxidizing archaeon from the open ocean. *PNAS* 2015; **112**: 1173–1178.

32. Ahlgren NA, Chen Y, Needham DM, Parada AE, Sachdeva R, Trinh V, et al. Genome and epigenome of a novel marine Thaumarchaeota strain suggest viral infection, phosphorothioation DNA modification and multiple restriction systems. *Environmental Microbiology* 2017; **19**: 2434–2452.

**Supplemental Tables**

**Table S1** Additional ammonia, nitrite, and nitrate measurements for shallow and bottom waters.

| Date | Depth (qualitative) | Depth (m) | Ammonium (micromolar) | Nitrite (micromolar) | Nitrate (micromolar) |
| --- | --- | --- | --- | --- | --- |
| 10/17/18 | Shallow | 2 | 0 | 6 | 16.77 |
| 10/17/18 | Bottom | 12 | 0 | 6.12 | 17.5 |
| 10/26/18 | Shallow | 2 | 0.94 | 5.63 | 14.19 |
| 10/26/18 | Bottom | 10 | 2.35 | 7.13 | 17.82 |
| 11/15/18 | Shallow | 2 | 0 | 7.35 | 15.28 |
| 11/15/18 | Bottom | 12 | 0.6 | 6.28 | 16.07 |
| 12/5/18 | Shallow | 2 | 0.1 | 7.78 | 27.2 |
| 12/5/18 | Bottom | 12 | 0.1 | 8.71 | 23.2 |
| 12/14/18 | Shallow | 2 | 4.11 | 7.14 | 29.84 |
| 12/14/18 | Bottom | 11 | 1.41 | 7.71 | 30.49 |
| 1/30/19 | Shallow | 2 | 7.3 | 0.86 | 28.55 |
| 1/30/19 | Bottom | 13 | 6.98 | 1.14 | 29.42 |
| 2/20/19 | Shallow | 1 | 9.55 | 0.93 | 24.7 |
| 2/20/19 | Bottom | 11 | 9.06 | 0.79 | 23.13 |
| 5/14/19 | Shallow | 2 | 2.47 | 1.14 | 5.29 |
| 5/14/19 | Bottom | 12 | 1.92 | 1.07 | 2.57 |
| 7/1/19 | Shallow | 1 | 1.8 | 1 | 3.93 |
| 7/1/19 | Bottom | 10 | 1.19 | 0.93 | 1.64 |
| 12/5/19 | Shallow | 2 | 11.65 | 3.04 | 15.88 |
| 12/5/19 | Bottom | 11 | 9.65 | 1.89 | 18.81 |

**Table S2** Metatranscriptome and metagenome reads recruited to MAG library.

| Sample | Transcripts (%) | Metagenome reads (%) |
| --- | --- | --- |
| 2018Oct17 (S) | 18.11 | 18.83 |
| 2018Oct17 (B) | 19.20 | 20.26 |
| 2018Oct26 (B) | 10.13 | 15.03 |
| 2018Nov15 (S) | NA | 24.77 |
| 2018Nov15 (B) | 18.56 | 24.45 |
| 2018Dec05 (B) | NA | 18.13 |
| 2019Feb20 (B) | 6.97 | 15.02 |
| 2019May14 (B) | 11.92 | 15.87 |
| 2019Jul01 (B) | 14.29 | 10.91 |
| 2019Dec05 (B) | 11.14 | 22.69 |

**Supplemental Figures**


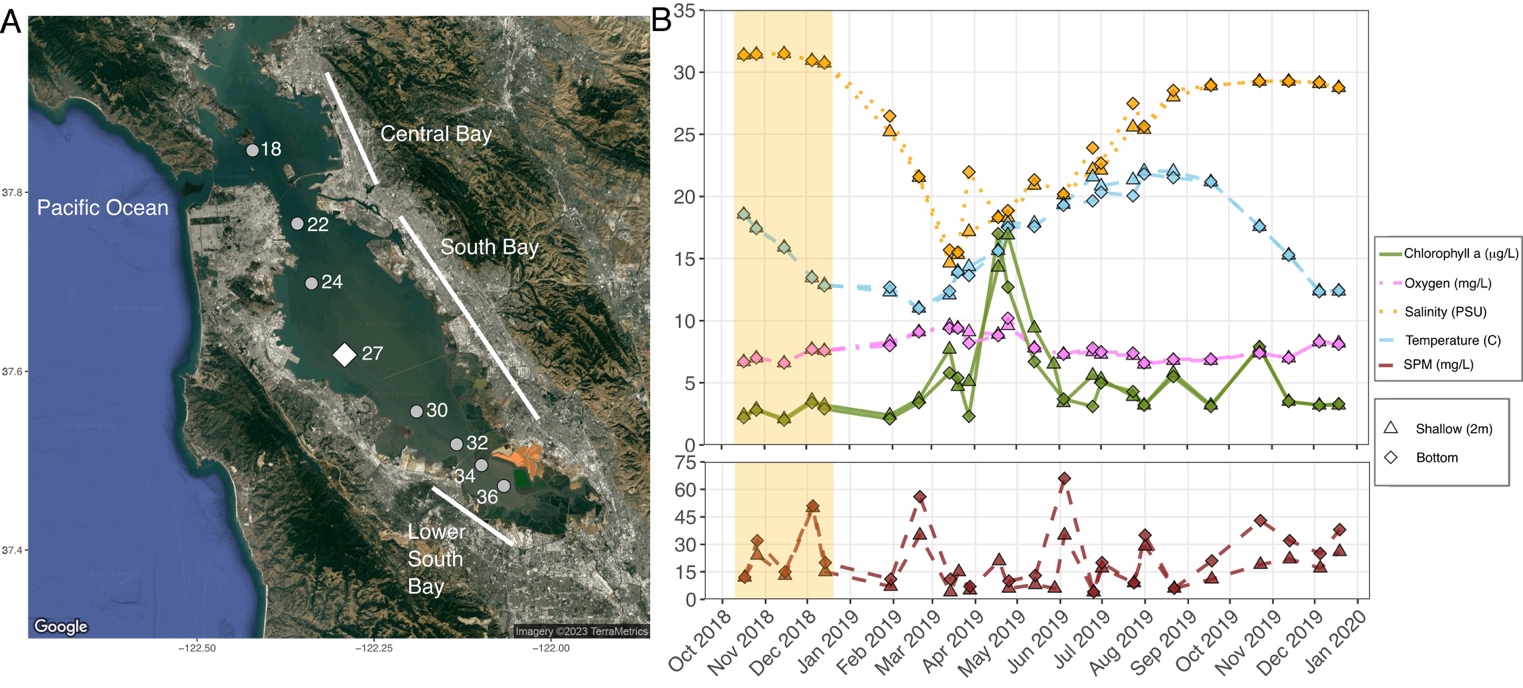


**Figure S1 A)** Map of South SFB USGS sampling stations indicated with grey circles, Station 27 highlighted in white diamond. B) Environmental variables for USGS Station 27 from October 2018 through December 2019 measured by USGS. Gold shading indicates AOA bloom samples.


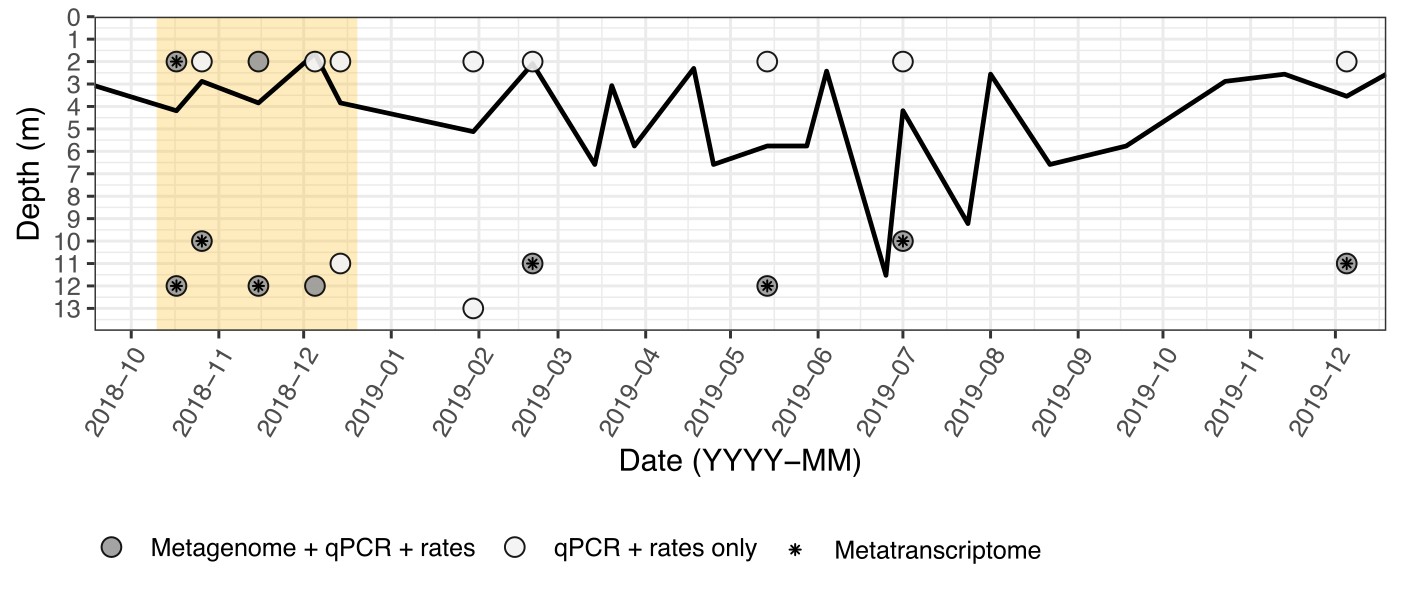


**Figure S2** Depth and dates of samples collected in this study indicated with circles, including shallow (2 m) and bottom water (1 m above the estuary floor) samples. Samples with metagenomes indicated by dark grey circles. Samples with metatranscirptomes indicated by asterisk. Solid black line indicates the photic depth (1% surface irradiance) based on the extinction coefficient measured by the USGS based on the formula I_Z_ = I_0_ e ^-k Z^ where I_0_ is the light intensity at the surface (100%), I_Z_ is the light intensity at the depth of interest (1%), e is the base of natural log, k is the extinction coefficient per meter, and Z is the depth in meters. Gold shading indicates AOA bloom samples.


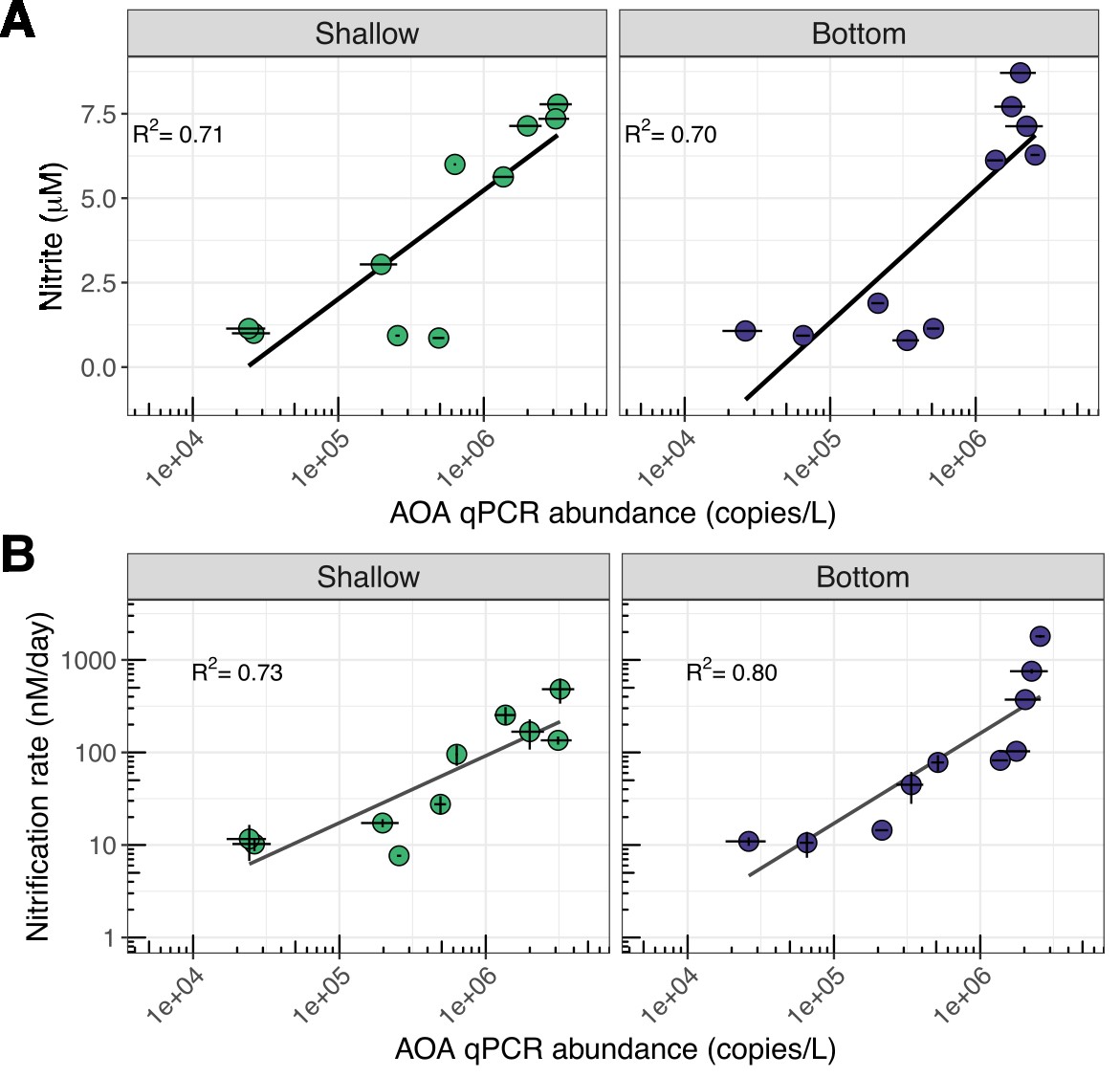


**Figure S3** A) Correlation between AOA qPCR abundance (copies of MGI 16S rRNA gene per L) in shallow and bottom water samples versus nitrite (A) and nitrification rates (B) with both axes on a log scale.

**Figure S4 A)** Visualization of pangenome of *Ca*. Nitrosomarinus catalina strains SPOT01, SFB27 (2013), and SFB27S_2018. **B)** Measures for allelic diversity in mapped reads compared to the representative (“reference”) genome as calculated in inStrain. Consensus ANI is shown in grey diamonds and measures the identity between mapped reads and the reference genome, calling a substituion when a position in the consensus (major) allele in mapped reads differs from the allele in the reference genome. Population ANI is shown in black circles and measures the identity between mapped reads and the reference genome, calling a substitution only when a base in the reference genome allele is not represented in either the major nor minor alleles.

**Figure S5** Transcript abundance for AOA genes of interest (y-axis) for the 8 metatranscriptome samples in chronological order (x-axis). Gene category assignment based in part on supplemental tables from [31] and [32] and UniProtKB gene names.

**Figure S6** Ratio of transcript abundance for relevant genes versus *amoA* transcript abundance and AMT2 versus AMT1 transcript abundance. Ratios versus AMT1 shown on a log scale due to low transcript abundance. Missing points indicate one or both genes had a transcript abundance of 0. Gold shading indicates AOA bloom samples.

**Figure S7** JGI-generated RNAseq data for select nitrifier genes. Values represent transcript abundance normalized to total community *rpoB* and *gyrA* transcript abundance. Each point represents transcript abundance for a gene on a different scaffold. Points are colored by the scaffold taxonomy assigned by JGI. Gold shading indicates samples from the AOA bloom. The y-axis is on a log scale.

**Figure S8** A) Concatenated ribosomal gene tree for *Nitrospinales* genomes made using concatenated amino acid sequences and IQ-TREE using model LG+R6 and 1000 bootstraps. B) An *nxrB* gene phylogeny based on MAFFT nucleotide alignment of gene fragments 1308 nt long using PhyML tree with 100 bootstraps. Both trees are midpoint rooted with nodes > 80% bootstrap support shown on tree and branch length scale shown by bar. MAGs from this study are in bold. C) Transcript abundance for genes of interest in putative NOB MAG.

**Figure S9** Transcript abundance for genes from all non-AOA and non-NOB MAGs related to N-uptake/utilization and coping with oxidative stress.

**Figure S10** A) PCoA ordination of MAGs based on RPKG abundance and using Bray-Curtis dissimilarity. Large, green circles represent metagenome samples, smaller points represent MAGs. MAG shape and color indicate how many genes have significant changes in transcript abundance with black triangles indicating MAGs with no genes with significant changes in transcript abundance between AOA bloom and non-bloom samples. B) Histogram of how many MAGs had a given number of genes with significant differential transcript abundance between AOA bloom and non-bloom metatranscriptome samples, excluding the AOA MAG which had over 600 genes with significant changes in transcript abundance.


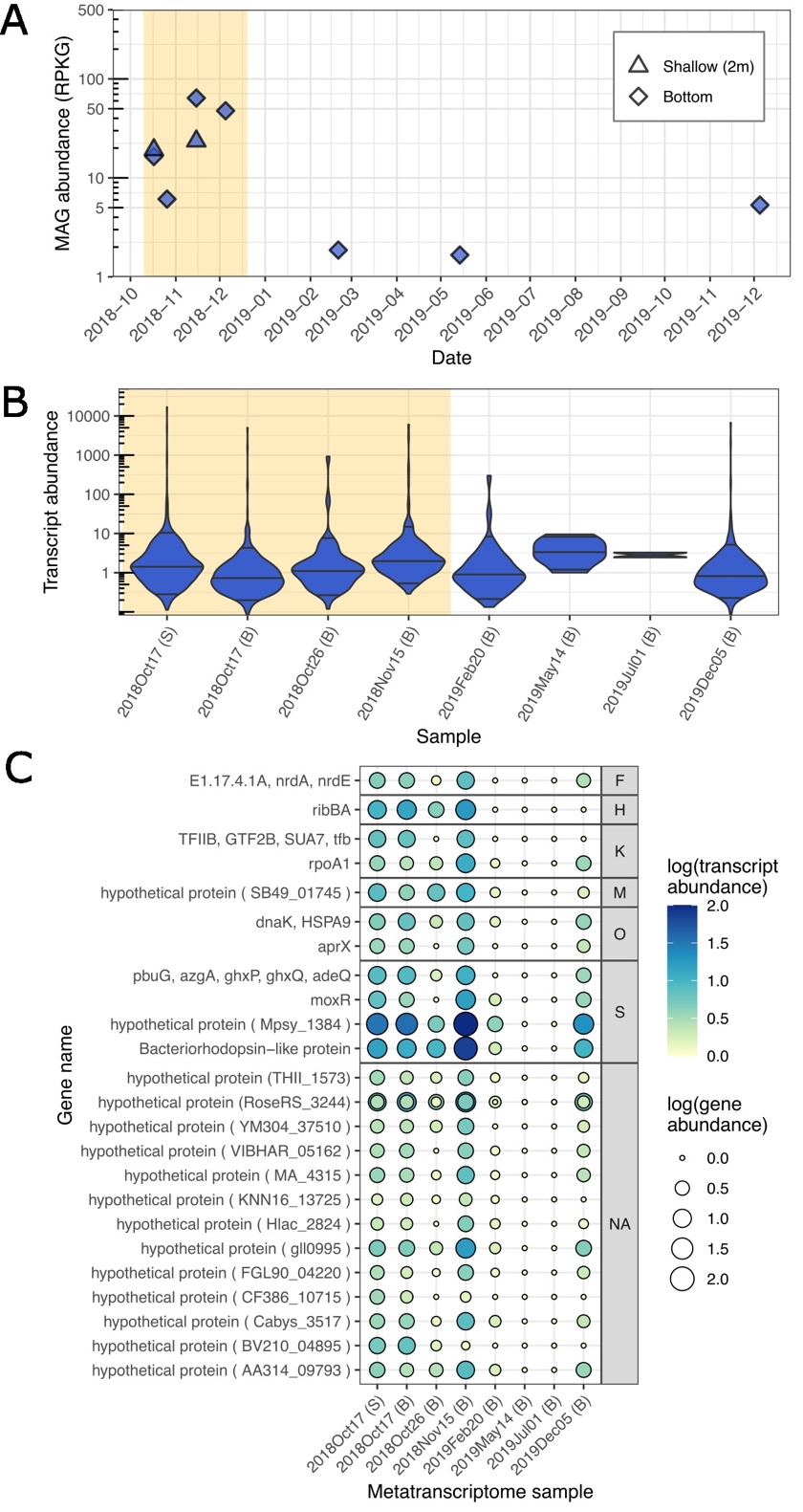


**Figure S11** A) Abundance of MGIIb-O2 genus MAG in metagenomes and B) transcript abundance represented by violin plots with constant width. Note y-axis is on a log scale. Golden highlights indicate AOA bloom samples. C) Genes with significant change between AOA bloom and non-bloom samples in transcript data. Genes (y-axis) include the ID and KEGG seed ortholog annotation if protein was hypothetical/unannotated. Panels are separated by COG functional category, including: Nucleotide transport and metabolism (F), Coenzyme transport and metabolism (H), Transcription (K), Cell wall/membrane/envelope biogenesis (M), Post-translational modification, protein turnover, chaperones (O), Function unknown (S) and unassigned functions (- or NA).
